# Supplementary material for: Clustering of the ζ-Chain Can Initiate T Cell Receptor Signaling
Source: Int J Mol Sci. 2020 May 15;21(10):3498. doi: 10.3390/ijms21103498 (PMC7279048; doi:10.3390/ijms21103498)
Supplement: Supplementary file 1 [file ijms-21-03498-s001.zip › ijms-802020-revision-suppl/ijms-802020-suppl.docx]

Movies:

Video 1 Representative movie of light induced clustering of LIC-Z in COS-7 cells. Cells are co-transfected with Lck10-GFP (left) and LIC-Z (right). Video were taken on LeicaSP5 confocal microscope using 63X/1.3 NA water objective under 594 nm laser excitation. The 458 nm laser was switched on briefly at 16s and 39s respectively to induce LIC-Z clustering. Fluorescence emission of Lck10-GFP and LIC-Z were collected between 470 – 540 nm and 590 – 670 nm two spectral bands. Scale bar=6 µm.

Video 2 Representative movie of light induced Ca2+ influx of Jurkat 76 cells co-transfected with LIC-Z and G-GECO. Channel shown are G-GECO (green), LIC-Z (red), and two channels merged. Video were taken on LeicaSP5 confocal microscope using 20X/1.0 NA water immersion objective under simultaneous excitation by 458 nm and 594 nm lasers. Fluorescence emission was collected between 470 – 540 nm and 590 – 670 nm two spectral bands. The unit of time stamp is in seconds. Scale bar = 400 µm.

Video 3 Representative two single cell movies of Zap70 tSH2 translocation from cell cytosol to plasma membrane upon light induced clustering of LIC-Z-YFP in the reconstituted COS-7 cells. Cells are co-transfected with LIC-Z-YFP, Lck GFP and Zap70 tSH2 three constructs showing in three channels from left to right. The video was taken on the Zeiss880 confocal laser scanning microscope using 63x 1.4 NA oil objective focusing at the middle of the cell. The unit of time stamp is in seconds. Scale bar = 10 µm.

Video 4 Representative Zap70 tSH2 translocation from cell cytosol to plasma membrane upon light induced clustering of LIC-Z-YFP in a population of reconstituted COS-7 cells. Cells are co-transfected with LIC-Z-YFP, Lck GFP and Zap70 tSH2 three constructs showing in three channels from left to right. The video was taken on the Zeiss880 confocal laser scanning microscope using 40x/1.1 NA water immersion objective focusing at the middle of the cell. The unit of time stamp is in seconds. Scale bar = 30 µm.

Video 5 Representative movie of Zap70 tSH2 translocate into newly formed LIC-Z clusters in reconstituted COS-7 cells. Cells are co-transfected with LIC-Z-YFP, Lck GFP and Zap70 tSH2 three constructs showing in three channels from left to right. Image taken by 63x 1.4 NA oil objective focusing at the basolateral membrane of the cell. The unit of time stamp is in seconds. Scale bar = 5 µm.
